# Supplementary material for: Complexome profiling on the Chlamydomonas lpa2 mutant reveals insights into PSII biogenesis and new PSII associated proteins
Source: J Exp Bot. 2021 Aug 26;73(1):245–62. doi: 10.1093/jxb/erab390 (PMC8730698; doi:10.1093/jxb/erab390)
Supplement: erab390_suppl_Supplementary_Dataset_S1 [file erab390_suppl_supplementary_dataset_s1.zip › Supplemental Dataset 1 - Excel List and all profiles/plots/MPPB_Cre12.g523850.html]

Trivial name: MPPB  
  
Euclidean distance: 1058838.90  
Mean Intensity (WT): 127671.52  
Mean Intensity (Mut): 125368.02  
Distance: 8.29  
  
MapMan: mitochondrial electron transport / ATP synthesis.cytochrome c reductase;protein.targeting.mitochondria  
  
p value of intensity sums Welch test: 0.9548
